# *Ab Initio* Thermochemistry of Highly Flexible Molecules for Thermal Decomposition Analysis

**DOI:** 10.1021/acs.jctc.3c00265

**Published:** 2023-06-13

**Authors:** Hyunguk Kwon, Giannis Mpourmpakis

**Affiliations:** Department of Chemical and Petroleum Engineering, University of Pittsburgh, Pittsburgh, Pennsylvania 15261, United States

## Abstract

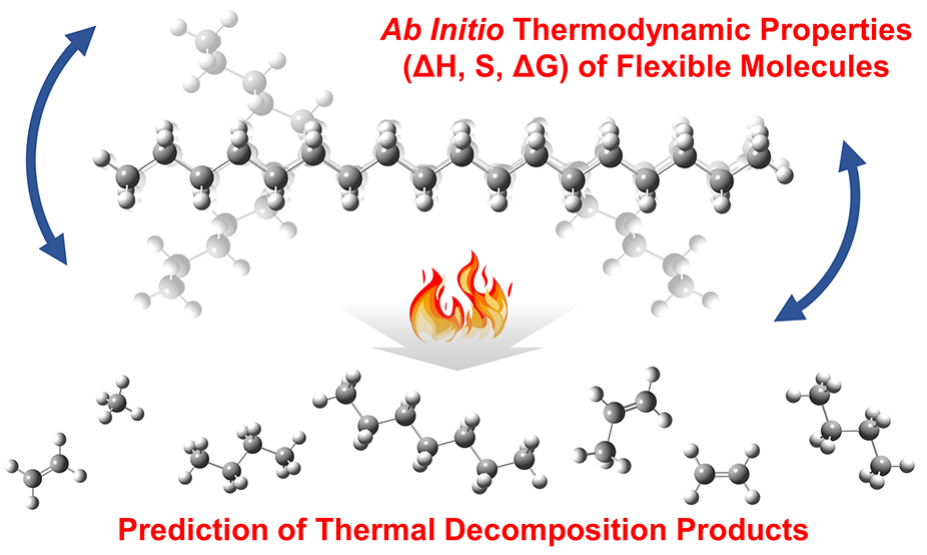

Pyrolysis is a promising technology for chemical recycling of waste plastics, since it enables the generation of high-value chemicals with low capital and operating cost. The calculation of thermodynamic equilibrium composition using the Gibbs free energy minimization approach can determine pyrolysis operating conditions that produce desired products. However, the availability of thermochemical data can limit the application of equilibrium calculations. While density functional theory (DFT) calculations have been commonly used to produce accurate thermochemical data (e.g., enthalpies of formation) of small molecules, the accuracy and computational cost of these calculations are both challenging to handle for large, flexible molecules, exhibiting multiple conformations at elevated (i.e., pyrolysis) temperatures. In this work, we develop a computational framework to calculate accurate, temperature-dependent thermochemistry of large and flexible molecules by combining force field based conformational search, DFT calculations, thermochemical corrections, and Boltzmann statistics. Our framework produces accurately calculated thermochemistry that is used to predict equilibrium thermal decomposition profiles of octadecane, a model compound of polyethylene. Our thermochemistry results are compared against literature data demonstrating a great agreement, and the predicted decomposition profiles rationalize a series of pyrolysis experimental observations. Our work systematically addresses entropic contributions of large molecules and suggests paths for accurate and yet computationally feasible calculations of Gibbs free energies. The first-principles-based thermodynamic equilibrium analysis proposed in this work can be a significant step toward predicting temperature-dependent product distributions from plastic pyrolysis and guide experimentation on chemical plastic recycling.

## 1 Introduction

The widespread production and use of plastics only date back to ∼1950, but nowadays plastics can be found everywhere in our daily life due to their low cost, light weight, and tunable properties. The global annual plastics production has reached approximately 380 million metric tons^1^ and is estimated to keep increasing over the next years.^2^ However, only ∼9% of all plastic waste is recycled,^3^ about 12% is incinerated, and the rest is discarded in landfills and the natural environment.^3^ The commonly used plastics derived from fossil fuels, such as petroleum and natural gas, are incredibly durable and not biodegradable. Therefore, plastic waste is expected to exist in landfills, oceans, and the environment in general for many decades before it decomposes.^4^ The poor management of plastic waste could be physically and chemically harmful to animals and public health and cause environmental pollution.^5,6^ In addition, incineration of plastic waste is a major source of air pollution by releasing toxic gases, such as dioxins, mercury, and halogens, into the atmosphere.^7^ Plastic waste is not only harmful to the ecosystem but also causes economic loss.^8^ Recycling plastic for reuse is cheaper than producing virgin plastics, with the monetary savings mostly arising from the energy savings.^9^ Each ton of recycled plastics can save up to ∼130 million kJ of energy, which is equivalent to the energy released by combustion of ∼22 barrels of oil.^9^ Thus, there is an urgent need to develop technologies that can efficiently recycle plastics of diverse compositions and reduce the serious economic loss and environmental problems.^4,10^

Several options exist for recycling of plastic waste.^11^ These are (1) reusing waste plastic products directly without altering them for the same purpose as the original plastic (primary or closed-loop recycling), (2) reprocessing waste plastic into secondary raw materials by physical means (secondary or mechanical recycling), (3) converting waste plastics to valuable chemical feedstocks or fuels (tertiary or chemical recycling), and (4) incinerating waste plastic to recover energy (quaternary recycling). Among them, the energy recovery through incineration is less favorable from an environmental point of view, and the energy generated in this way is substantially less than the energy conserved by recycling.^12^ The current recycling process heavily relies on the primary and secondary recycling.^9,13^ However, these techniques require a series of treatments and preparation steps, such as collection, separation, washing, and sorting of waste materials.^11^ To overcome the drawbacks, chemical recycling has been growing as an attractive route.^13^

Pyrolysis is one of the most practical and promising routes for chemical recycling of waste plastics.^14^ Pyrolysis technology can handle unsorted, unwashed plastics and is already being developed at a commercial scale.^15^ The main idea of pyrolysis technology is to obtain valuable chemicals produced by the decomposition of plastics in an oxygen-free, high-temperature environment. Although pyrolysis seems simple on its implementation, the yield and composition of pyrolysis products are controlled by many different operating conditions, such as temperature, pressure, reactor type, residence time, flow rate, and catalyst.^16^ Hence, the main challenge for realizing pyrolysis technology is to find optimal operating conditions for converting plastics into targeted high-value chemicals. Despite many investigations, selecting the optimal operating conditions heavily relies on trial-and-error experiments.

Thermodynamic modeling has been widely used to guide experiments involving complex chemical reactions, such as pyrolysis and gasification.^17−19^ The thermodynamic analysis predicts the chemical composition of a given system at the equilibrium state to aid in reactor design, unit operations, process optimization, and techno-economic and lifecycle analyses.^20,21^ Chemical equilibrium composition can be calculated in two ways:^18^ (1) stoichiometric method and (2) nonstoichiometric (or Gibbs energy minimization) method. The stoichiometric method uses the relationship between balanced chemical equations and equilibrium constants to determine equilibrium compositions. However, the set of potential chemical reactions considered are often chosen arbitrarily by chemical intuition, which greatly affects the resulting equilibrium composition.^22^ In addition, an accurate estimation of the initial equilibrium composition is necessary to avoid numerical divergence during computations.^23^ On the other hand, the Gibbs energy minimization method uses the concept that the Gibbs free energy of a system is minimized at thermodynamic equilibrium. In this approach, there is no need to specify a set of possible reactions *a priori*, as is required in the stoichiometric method. It only requires thermodynamic data as inputs, composition of reactants, and list of final products that are likely to be present at equilibrium. Thus, the Gibbs energy minimization method becomes more efficient and reliable for large chemical systems than the stoichiometric method.

While useful, there are some challenges with the Gibbs energy minimization method. First, finding a global minimum of an energy function demands a well-established optimization technique. Fortunately, this issue has been addressed in existing software, such as Chemkin,^24^ HSC chemistry,^25^ Aspen,^26^ Cantera,^27^ CEA,^28^ and CIRCE.^18^ Therefore, searching for a global minimum is doable unless we are solving a very complex system, such as a large chemical equilibrium system involving multiple phases and chemical species forming nonideal mixtures.^18^ The second challenge is that the availability of accurate thermochemical data used as input can limit the application of the Gibbs minimization method. In general, thermochemical data for an equilibrium simulation are taken from existing databases, such as the National Institute of Standards and Technology (NIST),^29^ the Active Thermochemical Tables (ATcT),^30^ the Burcat’s thermodynamic data,^31,32^ and the Design Institute for Physical Properties (DIPPR) 801.^33^ However, there has been a lack of accurate entropic data (and, thus, Gibbs free energy data) compared to data for enthalpy of formation of species.^34^ Empirical methods, such as group additivity,^35^ also offer the opportunity to predict thermochemical data of compounds that have not been reported in the literature. However, the empirical methods do not accurately represent large and complex organic molecules due to the lack of accounting for multiple conformational structures^36^ as well as steric and weak (i.e., dispersion) interactions.^37^ The empirical methods can also generate large estimation uncertainties for species involving functional groups that do not have rich data. Alternatively, for species the thermochemistry of which has been roughly estimated or has never been reported in literature, *ab initio* quantum chemistry methods can be applied. Specifically, density functional theory (DFT) has been proven to be a powerful tool to generate thermochemical data for equilibrium modeling, in combination with existing databases or empirical prediction methods. For example, Kraft and collaborators used *ab initio* thermochemistry to predict the equilibrium composition in the thermal decomposition of tetra-ethoxysilane^38^ and titanium tetra-isopropoxide.^39^ Swihart and Catoire also applied *ab initio* based thermochemical equilibrium analysis to Al-containing compounds to understand aluminum combustion.^40^ Despite such successes, the equilibrium simulation coupled with *ab initio* thermochemistry can be limited by the insufficient accuracy of DFT methods for extremely flexible, long-chain molecules like plastics or plastics-derived products. Popular DFT functionals can result in large systematic errors in the heat of formation and entropy even for *n*-alkanes, the most basic hydrocarbon molecules.^41,42^ In addition, highly accurate quantum mechanical methods are not applicable for large molecules due to the high computational cost.

The purpose of this work is 2-fold: to build and test a novel computational framework for polyethylene decomposition, as a first step toward plastics depolymerization simulations. First, we demonstrate the development of a computational framework for calculating the accurate temperature-dependent thermochemistry of extremely flexible molecules, by combining force field based conformational search, DFT calculations, empirical corrections on thermochemical data, and Boltzmann statistics. Classical molecular mechanics for conformational search followed by DFT calculation is a common approach in modeling flexible molecules. The lowest-energy conformer identified by a conformational search has been used as an input structure for DFT calculation in the literature.^43,44^ However, we show in this work that such a strategy may fail to calculate accurate thermodynamic properties of flexible molecules without consideration of various conformations. Second, as a proof-of-concept, we apply this framework to simulate the equilibrium product distribution from thermal decomposition of a polyethylene model compound using the calculated thermochemical data. Octadecane (C_18_H_38_), the smallest solid alkane at room temperature, is chosen as a model compound for polyethylene in this work, as long alkanes have been widely used as model compounds in literature.^45−48^ The *ab initio* thermochemistry-based equilibrium modeling proposed in this work can contribute to elucidating pyrolysis product distribution as a function of temperature at equilibrium and to selecting thermodynamically optimal operating conditions for the formation of desired, high-value products. Furthermore, the accurate *ab initio* thermodynamic properties can be potentially used in the development of a detailed kinetic model of large systems by incorporating kinetic data and predict product compositions addressing both thermodynamic and kinetic limitations.

## 2 Methods

**Figure 1:**
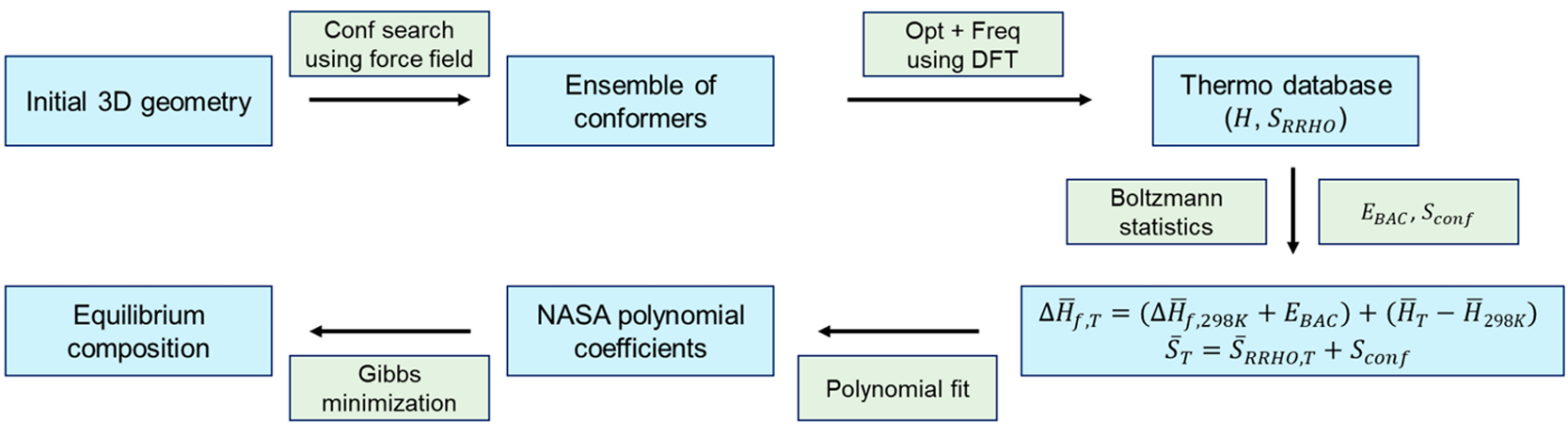
Workflow for *ab initio* thermochemistry calculation of flexible molecules and prediction of equilibrium composition. *H* and Δ*H*_*f*_ denote DFT-calculated absolute enthalpy and enthalpy of formation, respectively. *S*_*RRHO*_ and *S*_*conf*_ denote DFT-calculated entropy and conformational entropy, respectively. The notation of a straight bar above a letter ( ̅) denotes that the thermodynamic property is ensemble-averaged. *E*_*BAC*_ indicates bond additivity correction terms.

We built a computational framework for *ab initio* thermochemistry calculations of large, flexible molecules and equilibrium composition prediction, as shown in Figure 1. This framework is comprised by force-field-based conformational search, DFT calculations, thermochemical corrections, Boltzmann statistics, and Gibbs energy minimization. First, conformational search is performed based on force field energy minimization using the Global-MMX (GMMX) subroutine of PCMODEL.^49^ The tool searches the conformational space, based on a Monte Carlo search technique originally used in the BAKMDL program,^50^ by both randomly moving a set of heavy atoms in 3D Cartesian space and rotating randomly selected bonds in dihedral space.^51^ In our search, all rotatable bonds are designated for rotation. In a conformational search, the energy-minimized structure is compared against other previously energy-minimized structures to identify if the structure is unique. All structures within 0.1 kcal/mol of the current conformer are examined during structure comparisons, and geometrically similar molecules with a root-mean-square deviation below 0.25 Å of a conformer is discarded from the ensemble set. The benchmark simulations showed that the conformational search is not sensitive to the cutoff distance and energy parameters. The unique structure within a user-specified energy window is finally added to the ensemble set. Conformational search is performed once for a given structure, which is sufficient to refine input geometries for DFT calculations. The conformational search algorithm implemented in GMMX has been extensively used to identify conformations of highly flexible biomolecules consisting of more complex functional groups and hetero atoms than the organic molecules we focus on in this work.^52−54^ The MMFF94 force field^55^ implemented in PCMODEL is chosen, because it has shown good performance in the conformational analysis of organic molecules.^56^ After the conformational search, geometry optimization and harmonic frequency calculations are performed using Gaussian 09^57^ to obtain absolute thermodynamic properties of the identified conformers at the DFT level. For the DFT calculations, the M06-2x^58^ density functional along with the 6-31G(d) basis set are used. This functional is selected because it exhibits good performance for main group thermochemistry.^59,60^ While the M06-2x accounts for dispersion forces to some extent without a posterior fashion,^59,60^ the addition of dispersion corrections has been often recommended to further improve the functional’s performance.^61,62^ To evaluate the effect of dispersion corrections in our work, we calculated rigid-rotor-harmonic-oscillator (RRHO) entropy using the M06-2x-D3 for octadecane with a hairpin structure (see Figure S1) where the intramolecular dispersion forces between the folded chain segments could affect entropic calculations. The RRHO entropy difference between the M06-2x and the M06-2x-D3 was found to be only less than 0.5 cal/(mol·K). This benchmark calculation shows that the M06-2x functional without a dispersion correction is a suitable choice for this study. The enthalpy of formation at 298 K is derived using the atomization method.^63^ Errors in DFT energies have often been considered systematic, and the systematic errors can be canceled out using posteriori correction methods, such as the bond additivity correction (BAC),^64^ the atom additivity correction,^65^ and the probabilistic model derived correction.^65^ The BAC method is used in this work to reduce systematic DFT errors in the enthalpy of formation since it provides high chemical accuracy, is easy on its implementation, and is widely used in literature.^63,64^ To account for the entropic gain arising from different conformers, the conformational entropy is calculated and added to the DFT-computed RRHO entropy. In many computational chemistry studies, the lowest energy structure is used to calculate thermodynamic properties of a molecule and the ensemble of conformers is entirely neglected even for relatively flexible molecules.^66^ This approximation might be reasonable under certain conditions (e.g., at low temperatures), but it may limit the prediction of temperature-dependent thermodynamic properties with high accuracy. Therefore, each individual enthalpy of formation and entropy are weighted with respect to the Boltzmann probability of conformational isomers at each temperature to calculate the ensemble-averaged thermodynamic properties. After all the thermodynamic calculations, we store the temperature-dependent thermochemistry data as a seven-coefficient NASA polynomial using the Fitdat subroutine of Chemkin software.^24^ Finally, the thermodynamic data is used as input to predict equilibrium composition of a system using the Gibbs energy minimization approach. The equilibrium simulation is performed using Chemkin.

## 3 Results and Discussion

Αs a benchmark case, we applied the thermochemistry framework presented in Figure 1 to octadecane.

### 3.1 Conformational Search

We initially carried out conformational search using an all-trans (i.e., fully extended) structure as the initial structural configuration. However, the search failed to identify energetically important low-energy conformers. For example, a hairpin conformer with four gauche rotations,^67^ which is generally considered as the global minimum of flexible alkanes (C_*n*_H_2*n*+2_) with *n* ≥ 16–18 at low temperatures,^68−71^ was not identified by the conformational search. This result implies that the ensemble of conformers could be sensitive to the initial structure in the force field based GMMX conformational search. Thomas et al. also reported a similar behavior, where the internal coordinate Monte Carlo conformational searches using the OPLS-AA force field failed to identify hairpin structures for flexible C_14_H_30_–C_34_H_70_ alkanes.^69^ We therefore perform a two-stage conformational search to thoroughly identify energetically stable conformers. First, two independent conformational searches are carried out with two initial structures, all-trans and hairpin, to generate two groups of conformers. The two initial structures are chosen because they are energetically competing conformers of *n*-alkanes at low temperatures to be a global minimum depending on the chain length.^68,70^ The conformational searches for octadecane starting with all-trans and hairpin structures identify 500 and 4176 conformers, respectively, when an energy cutoff of 4 kcal/mol is used. Second, the two subgroups of structures are combined into one ensemble by discarding the duplicate conformers with the same energy. In the two subgroup sets, 60 duplicate conformers were found. After removing the duplicate conformers, 4616 unique conformers are finally included in the final ensemble. The probability to find the lowest energy conformers in the final ensemble using eq 5 was found to be 17% at 298.15 K. It should be noted that the potential energy, instead of the Gibbs free energy, is used to compute the probability, as calculation of enthalpy and entropy is not considered in a force-field based conformational search. This result shows that the two-stage conformational search enables more effectively sampling the conformational space than a typical conformational search using one structure.

Despite the thorough search, it was found that a force-field based conformational search fails to correctly identify the lowest-energy conformer of octadecane. The lowest-energy conformer in the investigated ensemble turns out to be the all-trans structure, however, which is not consistent with previous experiments^68^ and quantum mechanical calculations.^70^ It was reported that an all-trans conformation is the most favorable for short alkanes at low temperatures, whereas a hairpin conformation becomes more favorable from hexadecane (C_16_H_34_) to octadecane (C_18_H_38_) due to the intramolecular dispersion forces between the folded chain segments. As shown in Figure S1, however, the MMFF94 force field overestimates the transition point from an all-trans one to a hairpin one. At the MMFF94 force field level of accuracy, tetracosane (C_24_H_50_) is the first *n*-alkane in which the hairpin structure is energetically favored over the all-trans structure. The overestimation of the transition point has also been reported in previous papers with popular force-fields. The force-field-based transition point (number of carbon atoms) was found to be 18 for MM2,^67^ 25 for MM3,^67^ 26 for AMBER,^67^ and 22 for OPLS-AA.^69^ The poor prediction of the transition point by the force field methods partially arises from inaccurate descriptions of intramolecular dispersion forces.^72^ In summary, the force-field-based conformational search can be useful in generating a rich ensemble of conformers; however, it may not be appropriate to accurately identify a minimum-energy conformer of flexible molecules. To search for conformers with a higher accuracy, future studies could potentially apply metadynamics simulations combined with semiempirical tight-binding quantum chemistry (QC) methods.^42^ The tight-binding QC methods provide higher accuracy than classical force-fields, and the metadynamics algorithm accelerates conformational transitions with the biasing root-mean-square deviation (RMSD) potential. Currently this sampling approach would be suited to small to medium sized molecules (<100 atoms). However, it could become feasible to treat larger molecules with increasing computing power.

### 3.2 *Ab Initio* Thermochemistry Calculation

After the conformational search, we reoptimize the force-field geometries using DFT at the M06-2x/6-31G(d) level. The lowest-energy conformer of octadecane is found to be the all-trans one at the force field level as described above, whereas it is found to be the hairpin at the DFT level. The lowest-energy conformer predicted from DFT is consistent with previous experimental^68^ and computational^70^ studies. In addition, the transition point from the all-trans to the hairpin conformation is found to be at 16 carbon atoms in our DFT calculation (Figure S1), which is in good agreement with previous studies.^68,70^ This indicates that the M06-2x functional describes well the intramolecular dispersion forces between folded chain segments. Note that the M06-2x functional was trained using data sets that include noncovalent interactions, accounting for dispersion interactions.^58,60,73^ Following all geometry optimizations, (harmonic) frequency calculations are performed to calculate absolute thermodynamic properties (*H*_*i*_ and *S*_*RRHO*,*i*_) of each conformer *i*.

To accurately calculate the enthalpy of formation of flexible molecules with multiple conformers as a function of temperature, the enthalpy of formation of an isolated molecule at 298 K (Δ*H*_*f*,298*K*_) is first calculated using the atomization energy scheme,^63^ as follows:
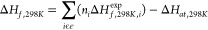
1where Δ*H*_*f*,298*K*,*i*_^*exp*^ indicates the experimental enthalpy of formation at 298 K of a gas phase atom of element *i*, taken from the literature.^74^*n*_*i*_ denotes the number of atoms of element *i* in the molecule. ε denotes the set of all elements (e.g., C and H for hydrocarbons). The theoretical atomization enthalpy at 298 K (Δ*H*_*at*,298*K*_) is given by
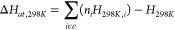
2where *H*_298*K*,*i*_ and *H*_298*K*_ denote the DFT-computed absolute enthalpy at 298 K of constituting atomic species *i* and the molecule of interest, respectively. Calculation of enthalpy of formation based on the atomization energy scheme is the most common and preferred method applicable to a large set of molecules.^63^ However, as shown in Figure S2, it generally introduces systematic errors, because atoms have significantly different electronic states than the closed-shell molecules.^75^ Alternatively, the isodesmic reaction has been used to yield more accurate enthalpy of formation, in which all formal bonds between non-hydrogen atoms are separated into the simplest diatomic molecules.^76^ This scheme uses as a reference a set of molecules, instead of a set of atoms, to reduce the systematic errors arising in the atomization energy scheme. However, as shown in Figure S2, the isodesmic reaction scheme exhibits more accurate predictions than the atomization scheme only for small hydrocarbons (up to C_4_ for alkanes and C_8_ for 1-alkenes). Figure S2 results indicate that the systematic errors in the calculated enthalpy of formation of large molecules would be unavoidable, as has been also observed in the literature.^63^ Hence, we use the atomization enthalpy method combined with the bond additivity correction (BAC) to cancel out the systematic errors^64^ as shown in eq 3.
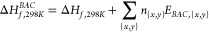
3

In eq 3, {*x*, *y*} is a pair of atoms (or a chemical bond) in a molecule, *n*_{*x*,*y*}_ is the number of bonds {*x*, *y*}, and *E*_*BAC*,{*x*,*y*}_ is a BAC correction parameter for the bond {*x*, *y*}. The BAC approach assumes the errors in calculated bond energies are constant for each type of bond. The BAC parameters are determined by least-squares fits to the enthalpy of formation for a set of molecules taken from the Burcat’s thermochemical database.^31,32^ Details on calculation of BAC parameters are given in Figure S3. Figure S3 shows that the enthalpy of formation corrected using the BAC method provides accuracy within 0.2 kcal/mol.

**Figure 2:**
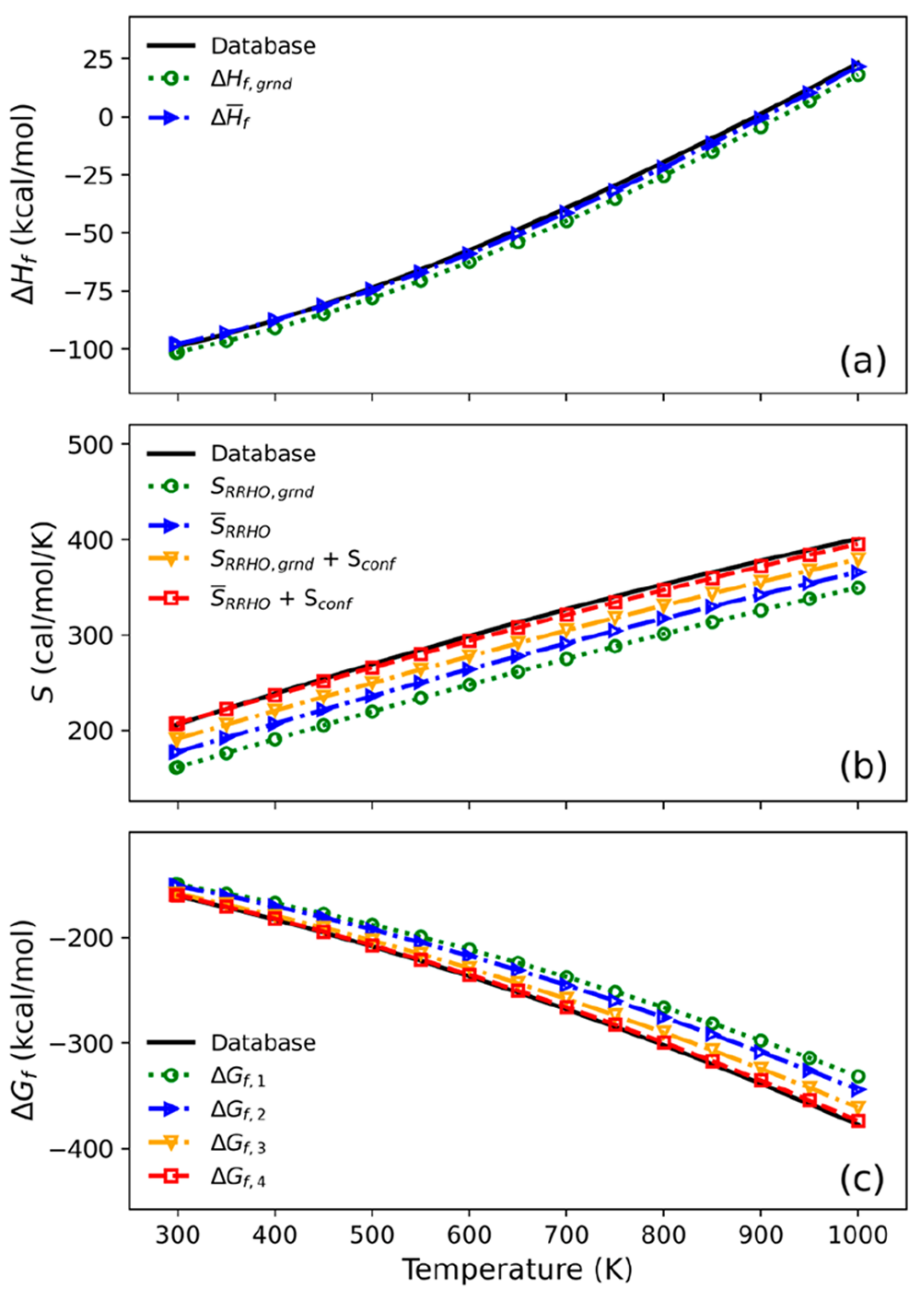
Temperature-dependent thermodynamic properties of octadecane calculated with DFT using different entropic formulations and accounting for Boltzmann statistics: (a) enthalpy of formation (Δ*H*_*f*_), (b) entropy (*S*), and (c) Gibbs free energy of formation (Δ*G*_*f*_). The subscript “grnd” denotes the thermodynamic property is calculated using solely the ground state energy conformer. The notation of a straight bar above a letter ( ̅) denotes that the thermodynamic property is calculated as an ensemble-averaged property. The different expressions of Δ*G*_*f*_ are presented in Table 1. Predictions are compared against Burcat’s thermochemical database.^31,32^

Thereafter, the ensemble-averaged thermodynamic properties of the molecules are calculated according to the Boltzmann probability (*p*_*T*,*i*_), using eqs 4 and 5.
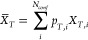
4with
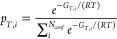
5where *X*_*T*,*i*_ denotes the thermodynamic properties, such as formation enthalpy, absolute enthalpy, and entropy, of a conformer *i*. *N*_*conf*_ is the number of conformers in the ensemble, *R* is the ideal gas constant, and *G*_*T*,*i*_ is the absolute Gibbs free energy of a conformer *i*. The ensemble-averaged thermodynamic property is computed using up to 300 low-energy conformers. In Figure S4, we tested the effect of the number of conformers on the ensemble-averaged property, and it turns out that 300 is sufficient to consider the ensemble effect. Finally, the ensemble-averaged enthalpy of formation at different temperatures (Δ*H̅*_*f*,*T*_) is calculated with the following formula.
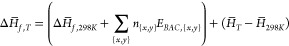
6This definition is referred to as engineering enthalpy of formation and has been used in the NASA polynomials.^77,78^Figure 2a compares the temperature-dependent DFT-computed enthalpies of formation of octadecane with values from the Burcat’s thermochemical database.^31,32^ When calculating the enthalpy of formation only using a minimum (ground state) energy conformer (Δ*H*_*f*,*grnd*_), the absolute deviation from the database is 2.4 kcal/mol at 298 K and 5.0 kcal/mol at 1000 K. By accounting for the ensemble effects using Boltzmann statistics, the absolute deviations at 298 and 1000 K are reduced to 1.1 and 1.4 kcal/mol, respectively. This result shows that calculating enthalpy of formation using an ensemble of conformers (Δ*H̅*_*f*_) improves chemical accuracy especially at higher temperatures, but the effect is not significant. This is especially important since calculating ensemble-averaged properties relies on an excessive number of DFT calculations which is a computationally intense task.

In contrast to enthalpy of formation, conformational variations can have stronger effects on entropy.^75,79^Figure S5 shows that the DFT-calculated RRHO entropies of *n*-alkanes with different basis sets systematically underestimate the entropy values from the database, when only a single (all-trans) conformer is considered. In addition, the underestimation increases with increasing molecular size, which implies that conformational effects could be a major part of this deviation. The partitioning of total entropy, first proposed by Karplus and co-workers,^80,81^ has been widely used to calculate entropy of flexible molecules at room and elevated temperatures in the literature.^42,82−84^ Assuming that a potential energy surface can be described as a collection of harmonic wells associated with different conformers, the total entropy can be calculated by the sum of the two, as follows.^82^
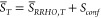
7where *S̅*_*RRHO*,*T*_ is the ensemble-averaged RRHO entropy that accounts for multiple energy wells of a flexible molecule. *S*_*conf*_ is the conformational entropy arising from conformational transitions among the energy wells, which (partially) accounts for effects of anharmonicities.^42,82,85^ The Gibbs–Shannon entropy formula (, where *p*_*i*_ is the Boltzmann probability and *R* is the ideal gas constant) is typically used to estimate the conformational entropy term.^42,83^ However, the accurate estimation of the Gibbs–Shannon entropy for large molecules is still very challenging, despite many efforts in the past. For example, Pracht and Grimme recently obtained conformational entropies of *n*-alkanes using a metadynamics driven search algorithm at the GFN-xTB (a semiempirical tight-binding quantum mechanical method) level of accuracy.^42^ However, their approach was applicable only up to hexadecane (*n*-C_16_H_34_) due to the increased computational cost. The main problem with estimating conformational entropy is that, in principle, one needs to calculate all possible conformers and their corresponding Gibbs free energies.^42^ However, the number of possible conformers can increase exponentially with the number of rotatable bonds and many low-energy conformers may be thermally accessible.^86^ Hence, instead of the Gibbs–Shannon entropy, we use an empirical formula to estimate conformational entropy by Ghahremanpour et al., who found that the contribution of conformational entropy per rotatable bond is close to the ideal gas constant^87^ (eq 8).
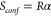
8In eq 8, *R* is the ideal gas constant and α is the number of rotatable bonds of a molecule. This empirical conformational entropy was applied to over 2000 compounds and allowed to greatly improve the accuracy of the DFT-calculated entropy.^87^Figure S6 also shows that the conformational entropy of *n*-alkanes estimated from the empirical formula is in excellent agreement with the literature values by Pracht and Grimme,^42^ with a deviation of less than 0.5 cal/(mol·K) on average. Figure 2b compares the DFT-calculated entropies with the entropy from the Burcat’s database for octadecane. The entropies are calculated through four different methods: (1) RRHO entropy with a minimum energy conformer (*S*_*RRHO*,*grnd*_), (2) ensemble-averaged RRHO entropy (*S̅*_*RRHO*_), (3) addition of *S*_*conf*_ to *S*_*RRHO*,*grnd*_ (*S*_*RRHO*,*grnd*_ + *S*_*conf*_), and (4) addition of *S*_*conf*_ to *S̅*_*RRHO*_ (*S̅*_*RRHO*_ + *S*_*conf*_). As shown in Figure 2b, if only a single minimum energy conformation is taken into account, the RRHO entropy (*S*_*RRHO*,*grnd*_) underestimates the entropy by 44.6 cal/mol (21.7%) at 298 K. Compared to *S*_*RRHO*,*grnd*_, *S̅*_*RRHO*_ shows a better prediction but is still underestimated by 28.5 cal/(mol K) (13.9%) at 298 K. When *S*_*conf*_ is added to *S̅*_*RRHO*_, the calculated total entropy (*S̅*_*RRHO*_ + *S*_*conf*_) is in very good agreement with the database values. The deviations are only 1.4 cal/(mol K) (0.7%) at 298 K and 5.3 cal/(mol K) (1.3%) at 1000 K. The slightly larger deviation at higher temperature might be because temperature effects are not considered in the empirical formula of *S*_*conf*_. According to Chan et al., conformational entropy of unbranched alkanes increases with temperature.^86^ This indicates that one may need to include temperature effects for highly accurate conformational entropy description, even though the temperature-independent conformational entropy assumed in this work still provides very good accuracy at elevated temperatures. Although *S̅*_*RRHO*_ combined with *S*_*conf*_ (*S̅*_*RRHO*_ + *S*_*conf*_) provides high accuracy, calculation of *S̅*_*RRHO*_ is the computational bottleneck because the application of the Boltzmann probability necessitates calculating the thermochemistry of each conformer in the ensemble using DFT. Therefore, we also calculate entropy through *S*_*RRHO*,*grnd*_ + *S*_*conf*_, which does not consider the Boltzmann distribution but the entropy of just the ground state conformer with the additional conformational entropy term. As shown in Figure 2, this entropy generates an error of 14.8 cal/(mol K) (7.2%) at 298 K, which is, unsurprisingly, less accurate than *S̅*_*RRHO*_ + *S*_*conf*_ but, surprisingly, more accurate than *S̅*_*RRHO*_. This result means that the conformational entropy caused by the degree of freedom of the different conformers (*S*_*conf*_) contributes to the total entropy more than the RRHO entropy change caused by the Boltzmann-averaged vibrational entropy contribution of the different conformers (*S̅*_*RRHO*_ – *S*_*RRHO*,*grnd*_).

Although the entropy partitioning approach based on eq 7 achieves accurate results, there is another well-known approach that computes the total entropy of flexible molecules. It explicitly accounts for anharmonic torsional modes arising from transition between low-lying conformations using hindered rotor (HR) models in one-dimensional or multidimensional formulations.^88^ The multidimensional treatment is not practical for large systems due to the high computational demands.^89^ On the other hand, the 1D HR model allows relatively large molecules to be treated under the assumption that an internal motion is not coupled with all other motions.^90^ Moreover, previous results showed that internal rotations of *n*-alkanes, alcohols, sulfides, and thiols^91,92^ can be well described within the 1D HR approach. Therefore, we calculate the entropy of all-trans octadecane using the 1D HR model and compare it with the entropy calculated using eq 7 (*S̅*_*RRHO*_ + *S*_*conf*_). For the 1D HR, relaxed scans of the dihedral angle of each alkyl group (C_*n*_H_2*n*+1_, where *n* = 1 to 9) are performed with a step size of 10 degrees using Gaussian 09 at the M06-2x/6-31G(d) level. The results are then used as input data for the calculation of the hindered rotor partition function using the python package TAMkin.^93^Figure S7 shows that the 1D HR model provides entropy (*S*_*HR*_) with a deviation of less than 4% at most from the Burcat’s database over all temperature ranges investigated. Compared to *S̅*_*RRHO*_ + *S*_*conf*_, *S*_*HR*_ is slightly less accurate at low to intermediate temperatures (<600 K) but comparably accurate at high temperatures (>600 K). Overall, both *S*_*HR*_ and *S̅*_*RRHO*_ + *S*_*conf*_ show high accuracy under the pyrolysis temperatures. However, even though accurate entropy can be obtained with the 1D HR model, it would not be suitable for a robust treatment of large molecules. Even for the relatively simple 1D model, substantial effort on identifying internal modes and obtaining torsional potential energy surfaces of each mode is required, which makes it unfeasible for a systematic first-principles thermochemistry workflow. Therefore, *S̅*_*RRHO*_ + *S*_*conf*_ values are used below to calculate Gibbs free energy of formation.

The enthalpy and entropy of formation are used to calculate Gibbs free energy of formation (Δ*G*_*f*_) of octadecane. Figure 2c shows Δ*G*_*f*_ as a function of temperature, and the equations used for Δ*G*_*f*_ calculations can be found in Table 1. As expected, ensemble-averaging improves chemical accuracy, but the improvement is not significant, considering that Δ*G*_*f*,2_ still shows a relatively large error (6% at 298 K and 9% at 1000 K). When conformational entropy is added to Δ*G*_*f*,2_, Δ*G*_*f*,4_ deviates from the database by less than 1% even at 1000 K. We also calculate free energy of formation with Δ*G*_*f*,3_ in which the thermochemistry of the minimum-energy conformer is used, and the conformational entropy is corrected. Interestingly, the chemical accuracy is satisfactorily improved at 298 K from 10.9 (Δ*G*_*f*,1_) to 2.0 kcal/mol (Δ*G*_*f*,3_) upon adding the conformational entropy, as shown in Table 1. However, the chemical accuracy of Δ*G*_*f*,3_ gradually decreases with temperature, and the error reaches up to 16.3 kcal/mol at 1000 K, which is not negligible. Overall, all these results demonstrate that Δ*G*_*f*,4_ provides the most accurate free energy of formation of flexible molecules (i.e., octadecane), but Δ*G*_*f*,3_ could be more practical since it exhibits very good accuracy (especially at relatively low temperatures) and much less computational cost.

**Table 1:** Different Expressions for Gibbs Free Energy of Formation of Octadecane

	Δ*G*_*f*_ (kcal/mol)		Deviation from database	
Equation	298 K	1000 K	298 K	1000 K
Δ*G*_*f*,1_ = Δ*H*_*f*,*grnd*_ – *TS*_*RRHO*,*grnd*_	–149.5	–331.5	10.9 (6.8%)	46.1 (12.2%)
Δ*G*_*f*,2_ = Δ*H̅*_*f*_ – *TS̅*_*RRHO*_	–150.7	–344.2	9.6 (6.0%)	33.4 (8.9%)
Δ*G*_*f*,3_ = Δ*H*_*f*,*grnd*_ – *T*(*S*_*RRHO*,*grnd*_ + *S*_*conf*_)	–158.3	–361.3	2.0 (1.3%)	16.3 (4.3%)
Δ*G*_*f*,4_ = Δ*H̅*_*f*_ – *T*(*S̅*_*RRHO*_ + *S*_*conf*_)	–159.6	–373.8	0.8 (0.5%)	3.8 (1.0%)
Burcat’s database	–160.4	–377.6	–	–

It should be noted that the proposed protocol has some limitations with system size. The conformational search based on classical force fields would not pose any restrictions, as it can be straightforwardly completed even on a common desktop computer. The computational bottleneck of our approach comes from the DFT calculations of each conformer to obtain the ensemble-averaged thermodynamic properties, as described above. At the M06-2x level of theory, geometry optimization and frequency calculation for one C_18_H_38_ conformer generally takes a few hours of computational time on high-performance supercomputers (on a CPU node with 24 cores). When the conformer calculations are parallelized, DFT calculations for an ensemble of conformers can be easily automated and completed within a few days. Hence, our approach would be applicable for molecules consisting of up to 100–200 atoms, depending on the user’s computational resources.

### 3.3 Equilibrium Composition in Octadecane Decomposition

**Figure 3:**
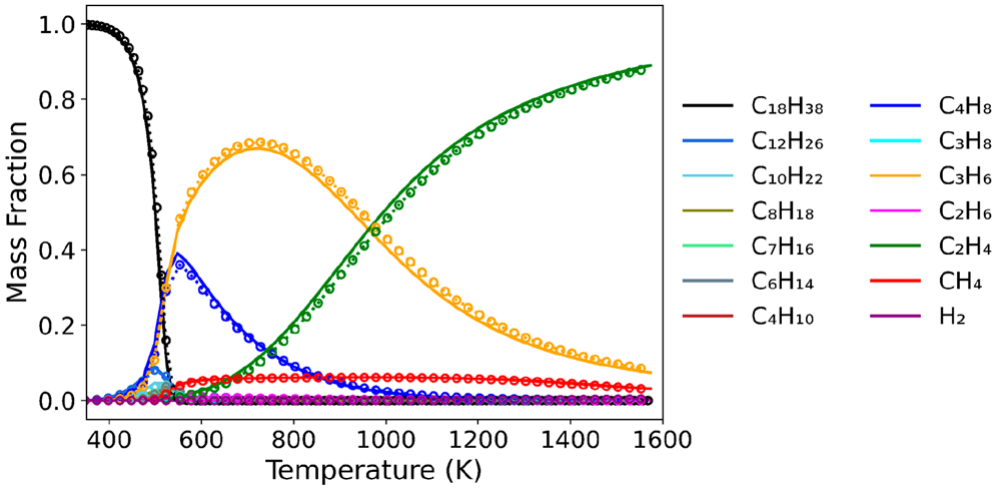
Equilibrium composition of octadecane decomposition using the Gibbs minimization method. Solid lines and dotted lines with circle markers denote the results using thermochemical data from the Burcat’s database and DFT, respectively.

The accurately calculated Gibbs free energy of formation can be utilized to predict equilibrium composition in a multicomponent mixture system using the Gibbs free energy minimization approach. At fixed temperature and pressure, chemical equilibrium is reached when the total Gibbs free energy of a system (*G*_*sys*_) is minimized. The total Gibbs free energy of a mixture can be expressed as the sum of the Gibbs free energies of formation of each species plus the Gibbs free energy of mixing that can be approximated with the ideal gas mixing term, as shown in eq 9.
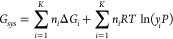
9where *n*_*i*_ and *y*_*i*_ represent the number of moles and mole fraction of species *i* in the system, respectively, and *P* indicates pressure. In the Gibbs energy minimization method, the objective is to determine the set of *n*_*i*_’s that minimize the value of *G*_*sys*_, and *n*_*i*_ needs to satisfy the elemental mass balance as a constraint. In our equilibrium simulation, octadecane is considered as the reactant. We consider the possible products from octadecane decomposition to be hydrogen (H_2_), methane (CH_4_), ethane (C_2_H_6_), ethylene (C_2_H_4_), propane (C_3_H_8_), propylene (C_3_H_6_), butane (C_4_H_10_), 1-butene (C_4_H_8_), hexane (C_6_H_14_), heptane (C_7_H_16_), octane (C_8_H_18_), decane (C_10_H_22_), dodecane (C_12_H_26_), and octadecane (C_18_H_38_). Hydrogen and C_1_–C_4_ alkanes and alkenes are typical gaseous products formed by thermal decomposition of hydrocarbon fuels.^94^ C_6_–C_12_ alkanes are components of gasoline or jet-fuels. For hydrogen and C_1_–C_3_ species, the free energy of formation is calculated using a global-minimum structure, since the conformational effects are not significant. On the other hand, the free energies of formation of C_4+_ species with multiple conformers are calculated using the approach proposed in Figure 1. The calculated thermochemical data for all species are presented in the Supporting Information (Figures S8 and S9). Figure 3 shows the calculated equilibrium composition of octadecane decomposition at 1 atm pressure and temperature ranging from 323 K (50 °C) to 1573 K (1300 °C) using the Gibbs free energy of formation data from Δ*G*_*f*,4_ equation and the Burcat’s database. For clarity, the same data focusing on the temperature range from 400 to 600 K (overlapping plots) are shown in Figure S10. The equilibrium compositions calculated using the thermochemical data from the two different sources (DFT vs Burcat) agree very well with each other. This demonstrates that the thermochemistry framework described in Figure 1 yields highly accurate thermochemical data of flexible molecules with multiple conformers. Additionally, we calculate equilibrium compositions using Δ*G*_*f*,1_, Δ*G*_*f*,2_, and Δ*G*_*f*,3_ data (Figure S11). All results show some deviations from the reference (database results), but the deviation decreases going from Δ*G*_*f*,1_, to Δ*G*_*f*,2_, to Δ*G*_*f*,3_. Therefore, considering the trade-off between computational cost and accuracy, one may be able to apply Δ*G*_*f*,3_ formulation for computationally expensive, large systems with excessive conformations.

Figure 3 shows that octadecane is thermodynamically limited to decompose below 450 K. Decomposition starts around 450 K and completely converts to small products at 550 K. The major species produced from the octadecane decomposition are propylene (C_3_H_6_) and 1-butene (C_4_H_10_). These two species are the most important products up to 720 K. However, in the temperature range of 550–720 K, the mass fraction of 1-butene gradually decreases, while that of propylene keeps increasing. The most dominant species is propylene for temperatures in the range 720–1000 K and then ethylene above 1000 K. The mass fractions of alkanes and hydrogen are negligible at all investigated temperatures. Methane is the only non-negligible alkane product with a mass fraction of approximately 0.05 above 600 K. While many experimental pyrolysis studies on polyethylene have been conducted (and could potentially make some connections to the octadecane model compound of our study), it is challenging to quantitatively compare product distributions in experiments because of differences in operating conditions.^95^ Nevertheless, some general trends for product distribution have been derived from previous pyrolysis studies. At higher temperatures or longer residence time, the production of lighter gas-phase products increases.^94,96^ Also, the concentrations of ethylene and propylene, as well as the alkene-to-alkane ratio for C_2_ and C_3_ increase with increasing pyrolysis temperature.^94,97^ At very high temperatures (around 1000 K), the major gas products are methane, ethylene, and propylene, with the highest concentration of ethylene.^95^ All these trends are in great agreement with our equilibrium simulations. This indicates that equilibrium simulations coupled with DFT calculations can potentially provide critical knowledge to guide the selection of operating pyrolysis conditions. Although such information is solely based on thermodynamics and does not incorporate any kinetic data, it is highly valuable to identify ideal reaction conditions (e.g., temperatures where kinetics are assumed to be accessible and there is only thermodynamic control in the reactions) that lead to the formation of desired products.

Despite the equilibrium simulation presented in Figure 3 assumes all species to be in the gas phase (single phase), some species may be in the liquid phase at the temperature conditions studied. Specifically, boiling points of species containing 6 or more carbons lie in the temperature range of 324 K–590 K, which is in the temperature ranges we investigate. To identify how much the equilibrium composition could change considering liquid phase species, we perform equilibrium simulations with a two-phase (gas–liquid) mixture system. Ideal behavior is modeled for both vapor and liquid, because low pressure (1 atm) and high temperature (50–1300 °C) conditions are simulated, and the system only contains nonpolar molecules with weak interactions with each other. Aspen^26^ is used in the multiphase equilibrium simulation. All gas and liquid thermochemical data are taken from the NIST database^98^ that is embedded in Aspen. As presented in Figure S12, below 500 K, liquid octadecane is uniquely present and no other species are present in both gas and liquid phases. Liquid octadecane decreases rapidly above 500 K with a simultaneous increase of 1-butene and propylene in the gas phase. When comparing the single- and two-phase results, the reactant (octadecane) decomposition profile slightly changes with temperature due to the different thermodynamic stability of liquid and vapor octadecane. However, the product distribution appears to be relatively unchanged (Figure S12) between the two simulations, because the major products are formed only at the vapor phase. Thus, we conclude that the assumption that all species are present in the single (gas) phase is valid in the equilibrium simulation of the system we investigate, particularly at high temperature conditions.

**Figure 4:**
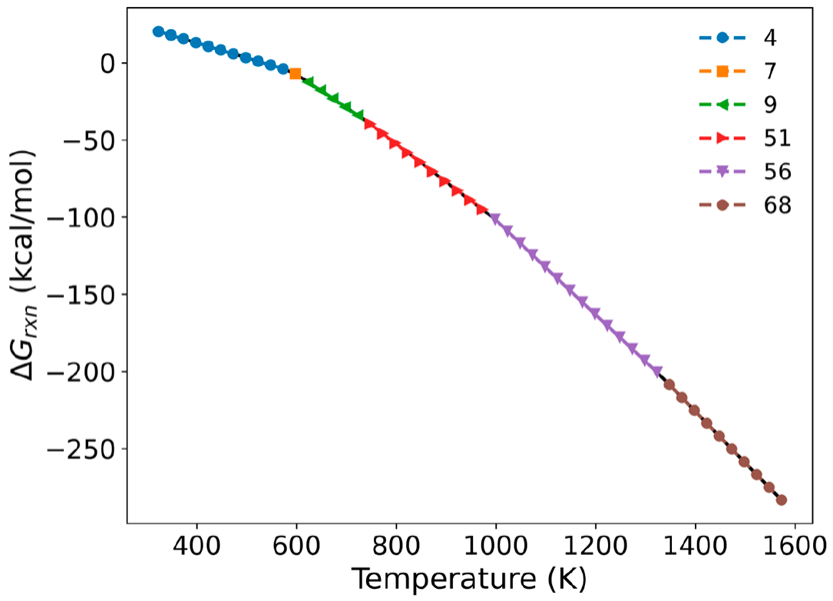
Octadecane decomposition reactions that show minimum Gibbs free energy at different temperatures. The reaction indices denoted in the legend can be found in Table 2.

Lastly, we calculate the Gibbs free energy of reactions (Δ*G*_*rxn*_) involved in octadecane decomposition to identify which decomposition reactions majorly determine the equilibrium composition at each temperature. In general, thermal decomposition of long-chain hydrocarbons begins through homolytic C–C bond scissions, and subsequent reactions, such as β-scission or H-abstraction, stabilize hydrocarbon radicals to form low-molecular weight saturated and unsaturated products.^99^ We first identify all possible overall reactions directly forming the saturated and unsaturated products in octadecane decomposition (depending on the products we consider), and the number is found to be 68. The list of the identified reactions is tabulated in Table S1, and the Gibbs free energies for all the reactions are shown in Figure S13. Figure 4 shows the minimum Gibbs free energy of reactions at each temperature, and the reaction lists are tabulated in Table 2. The thermodynamically most favorable reaction is found to be C_18_H_38_ → 2C_3_H_6_ + C_12_H_26_ below 573 K. However, at this low temperature, the reaction is almost thermoneutral, indicating an equilibrium between the octadecane and decomposition products. This result explains why octadecane begins to actively decompose at 450–550 K in the equilibrium simulation shown in Figure 3. Around 600 K, propylene is dominantly produced together with a relatively large alkane, hexane. As the temperature increases, propylene is produced along with smaller hydrocarbons, such as propane, methane, and ethylene. At even higher temperatures (>973 K), ethylene is the most favorable species. These results explain why (1) light alkene products are formed at high temperatures and (2) the alkene-to-alkane ratio increases with temperature in experiments^94,97^ (based also on the equilibrium simulation of Figure 3). At elevated temperatures the entropic contribution (−*T*Δ*S*) of the reactions takes over favoring smaller unsaturated complexes, like ethylene.

**Table 2:** Most Thermodynamically Preferred Reactions of Octadecane Decomposition at Different Temperature Regimes

Reaction index	The most preferred reactions	Temperature regime (K)
4	C_18_H_38_ → 2C_3_H_6_ + C_12_H_26_	313–573
7	C_18_H_38_ → 4C_3_H_6_ + C_6_H_14_	598
9	C_18_H_38_ → C_3_H_8_ + 5C_3_H_6_	623–723
51	C_18_H_38_ → CH_4_ + C_2_H_4_ + 5C_3_H_6_	748–973
56	C_18_H_38_ → CH_4_ + 7C_2_H_4_ + C_3_H_6_	998–1323
68	C_18_H_38_ → H_2_ + 9C_2_H_4_	1348–1573

The first-principles-based thermal decomposition framework introduced herein is based on thermodynamic equilibrium and does not account for any kinetic descriptions. However, it is powerful to use to identify reaction conditions where desired reactions will be thermodynamically accessible and undesired reactions, inaccessible. Most importantly, it is a framework that can be entirely based on DFT calculations, and thus, has predictive power on guiding experimentation. This is especially important in complex reaction networks (e.g., depolymerization) where predicting thermodynamic product distribution can be challenging. Computational frameworks like the ones presented herein can further feed microkinetic models where catalytic performance kinetic data can be accessible either through computations or experimentation, so detailed, temperature-dependent catalytic profiles can be developed taking into consideration thermodynamic and kinetic equilibrium calculations.

## 4 Conclusions

In this work, we introduced a computational framework to simulate chemical equilibrium of thermal decomposition of flexible molecules using thermochemistry based on first-principles calculations. First, we generated an ensemble of conformers using force-field-based conformational search and then calculated the thermochemistry of each conformer using DFT. We presented a procedure for the accurate calculation of formation enthalpy and entropy of flexible molecules. The DFT-calculated enthalpy of formation was corrected using an empirical correction, BAC, to reduce systematic errors. To account for accurate entropic contributions between different conformers, the conformational entropy was considered in addition to the RRHO entropy. The enthalpy of formation and entropy were ensemble-averaged according to the Boltzmann distribution to consider the effect from conformer ensembles. Accurate thermochemical data were calculated for octadecane, a model compound for polyethylene, and inputted in Gibbs free energy minimization to calculate equilibrium composition of thermal decomposition. We demonstrated an excellent agreement between compositions calculated using DFT-thermochemical data and an existing thermochemical database. The yielded equilibrium composition rationalized the experimentally observed product distributions of polyethylene pyrolysis. The DFT-based equilibrium simulation framework for large, flexible molecules proposed in this work can provide thermodynamic guidance for selecting pyrolysis operating conditions to decompose large hydrocarbons (e.g., plastics) to target, high-value chemicals.

## Supporting Information Available

Detailed energy information and thermochemical properties (enthalpy of formation and entropy) of hydrocarbon species, property comparison with database, ensemble averaged properties, thermochemical data in NASA polynomial format, and equilibrium compositions (PDF)
Geometries of all conformers (TXT)
