# Supplementary material for: Ab Initio Thermochemistry of Highly Flexible Molecules for Thermal Decomposition Analysis
Source: J Chem Theory Comput. 2023 Jun 13;19(12):3652–63. doi: 10.1021/acs.jctc.3c00265 (PMC10308812; doi:10.1021/acs.jctc.3c00265)
Supplement: Supplementary file 1 — ct3c00265_si_001.pdf [file ct3c00265_si_001.pdf]

Supporting Information

Ab Initio Thermochemistry of Highly Flexible Molecules for Thermal Decomposition Analysis

Hyunguk Kwon and Giannis Mpourmpakis*

Department of Chemical and Petroleum Engineering, University of Pittsburgh, Pittsburgh, Pennsylvania 15261, United States

**Corresponding author: gmpourmp@pitt.edu*

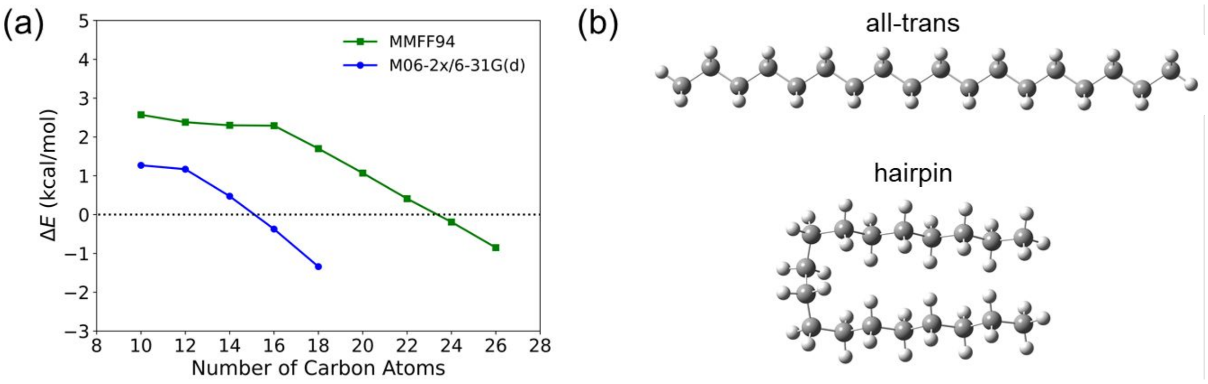

Figure S1. (a) The energy difference (ΔE) between all-trans and hairpin structures of *n*-alkanes calculated from force field (MMFF94) and DFT (M06-2x/6-31G(d)) methods. The energy difference is calculated by $E_{\text{hairpin}} - E_{\text{all-trans}}$. (b) Examples of all-trans and hairpin structures for octadecane ($C_{18}H_{38}$) optimized with DFT.

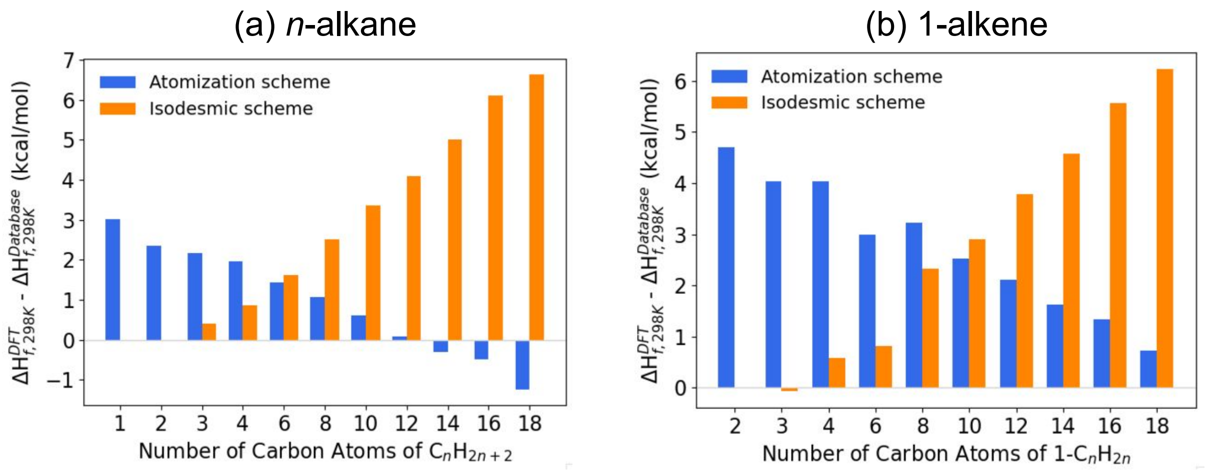

Figure S2. Difference between DFT-calculated enthalpy of formation ($\Delta H_{f,298K}^{DFT}$) and enthalpy of formation from the Burcat's database ($\Delta H_{f,298K}^{Database}$)[1,2] for (a) *n*-alkane and (b) 1-alkene. The DFT-calculated enthalpies of formation are calculated using the atomization scheme and the isodesmic scheme. All DFT calculations are performed using the all-trans structures.

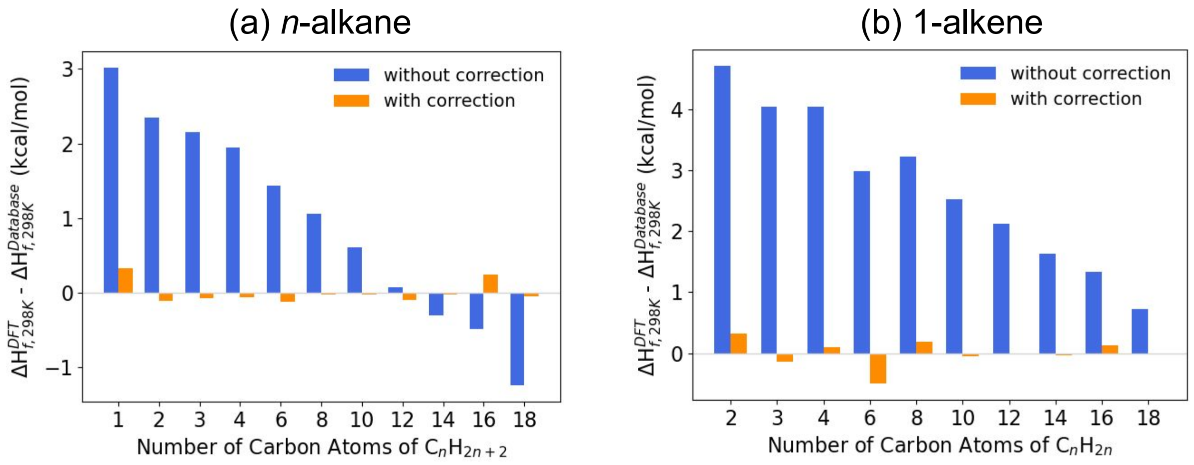

Figure S3. Difference between DFT-calculated enthalpy of formation ($\Delta H^{DFT}_{f,298K}$) and enthalpy of formation from the Burcat's database ($\Delta H^{Database}_{f,298K}$) for (a) *n*-alkane and (b) 1-alkene. The DFT enthalpy of formation is calculated using the atomization scheme without and with bond-additivity correction (BAC).[3] 11 alkanes and 10 alkenes shown in Figure S3, and hydrogen are used as reference species to calculate BAC parameters for C-C, C=C, C-H, and H-H bonds. In the BAC procedures, all enthalpies of formation are calculated using the all-trans structures. The calculated BAC parameters are 1.572 for C-C, -1.710 for C=C, -0.672 for C-H, and -2.877 for H-H.

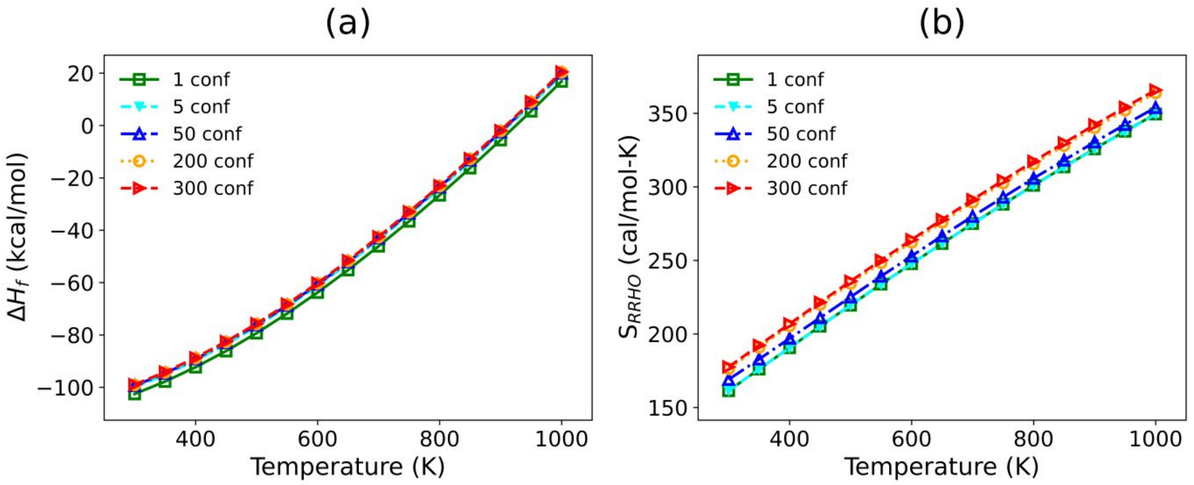

Figure S4. Effect of the number of conformers on ensemble-averaged enthalpy of formation and entropy. All results are calculated using DFT (M06-2x/6-31G(d)).

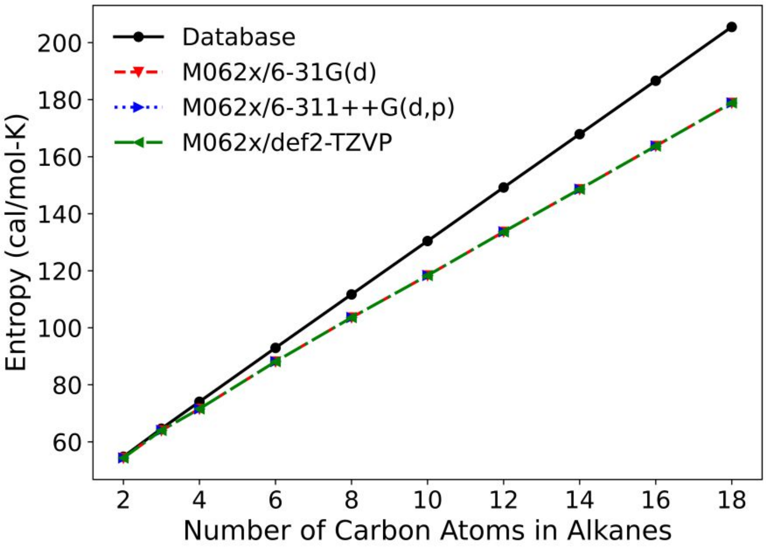

Figure S5. DFT-calculated entropy using the M06-2x density functional with different basis sets vs. entropy from the Burcat's database for *n*-alkanes. The DFT calculations are performed with all-trans *n*-alkanes.

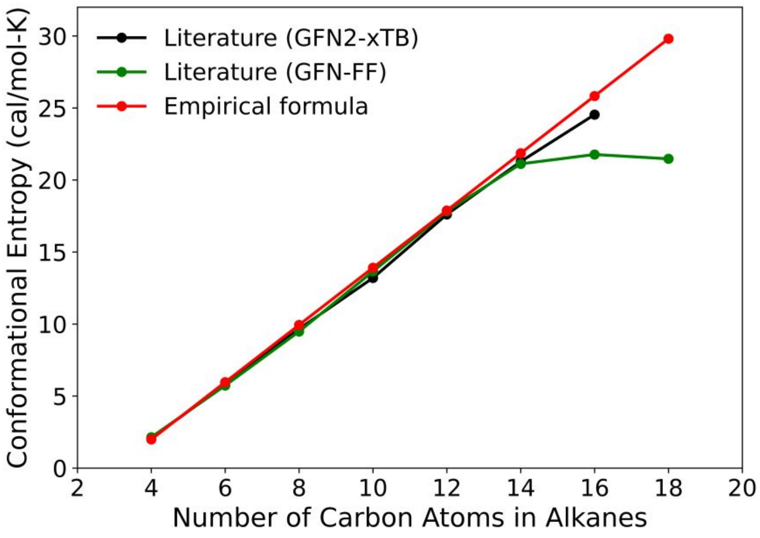

Figure S6. Conformational entropies calculated from the empirical formula from literature. The empirical formula for conformational entropy ($S_{conf} = R\alpha$, where R is the ideal gas constant and α is the number of rotatable bonds of a molecule) is taken from prior work by Ghahremanpour *et al.*,[4] and the values are plotted in red. The reference values plotted in black and green are extracted from Pracht and Grimme.[5]

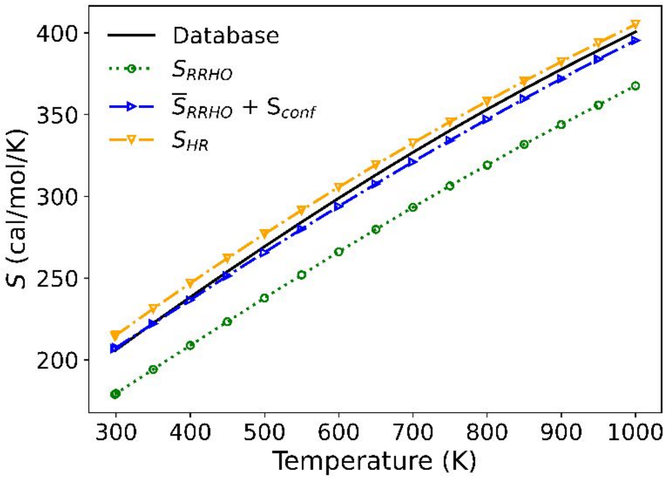

Figure S7. DFT-calculated entropy with different methods vs. entropy from the Burcat's database for octadecane ($C_{18}H_{38}$). S_{RRHO}, $\bar{S}_{RRHO}$, and S_{HR} indicate RRHO (rigid rotor harmonic oscillator) entropy, ensemble-averaged RRHO entropy, and entropy corrected with HR (hindered rotor) model, respectively. S_{conf} indicates conformational entropy. The DFT calculations are performed with an all-trans structure using M06-2x/6-31G(d).

```
THERMO ALL
   200.000  1000.000  2000.000
H2                      C   0H   2          G   200.000  2000.000 1000.00      1
 4.08785013E+00-1.90976175E-03 2.07260922E-06-7.98969486E-10 1.10913922E-13    2
-1.18018347E+03-7.21551107E+00 3.47705799E+00 2.06911797E-04-6.12658575E-07    3
 6.64714027E-10-1.73383202E-13-1.04170029E+03-4.18716831E+00                   4
CH4                     C   1H   4          G   200.000  2000.000 1000.00      1
 3.70477107E+00 2.33091236E-03 5.50535705E-06-3.93137149E-09 7.66509892E-13    2
-1.02211665E+04-7.42154002E-03 4.74951136E+00-9.59350124E-03 3.50101561E-05    3
-3.13466900E-08 9.55670263E-12-1.00428419E+04-3.11132639E+00                   4
C2H6                    C   2H   6          G   200.000  2000.000 1000.00      1
 6.04175649E+00 5.56513440E-03 8.09145006E-06-6.36540290E-09 1.28780315E-12    2
-1.27456699E+04-1.01820089E+01 3.65393524E+00-5.31561022E-04 4.07084638E-05    3
-4.37580591E-08 1.45479623E-11-1.14857067E+04 5.24984341E+00                   4
C2H4                    C   2H   4          G   200.000  2000.000 1000.00      1
 7.35744617E+00-1.96718771E-03 1.11686365E-05-6.82034382E-09 1.28481867E-12    2
 3.76749371E+03-1.66575356E+01 2.94943498E+00 1.87595596E-03 2.60872726E-05    3
-3.05550023E-08 1.06657086E-11 5.33854101E+03 8.05579560E+00                   4
C3H8                    C   3H   8          G   200.000  2000.000 1000.00      1
 8.27410940E+00 9.78299339E-03 9.27023524E-06-8.05441177E-09 1.67282880E-12    2
-1.63432028E+04-1.99204761E+01 3.62766158E+00 6.35408834E-03 4.74356373E-05    3
-5.55127095E-08 1.90410773E-11-1.43131785E+04 7.99962281E+00                   4
C3H6                    C   3H   6          G   200.000  2000.000 1000.00      1
 8.09687672E+00 6.47095822E-03 7.92539811E-06-6.48496476E-09 1.32872071E-12    2
-1.05513620E+03-1.80529183E+01 2.67531332E+00 1.13956957E-02 2.56805661E-05    3
-3.50832596E-08 1.26686735E-11 8.67252285E+02 1.22933702E+01                   4
C4H10                   C   4H  10          G   200.000  2000.000 1000.00      1
 1.06636091E+01 1.35806552E-02 1.08681622E-05-9.92817823E-09 2.08813474E-12    2
-1.99303775E+04-3.05303904E+01 3.87012899E+00 1.21023779E-02 5.60638746E-05    3
-6.87108508E-08 2.39468523E-11-1.71390716E+04 9.40727337E+00                   4
C4H8                    C   4H   8          G   200.000  2000.000 1000.00      1
 1.03101628E+01 1.07215577E-02 9.04498656E-06-8.13599440E-09 1.70597261E-12    2
-4.47705661E+03-2.79073268E+01 2.65747777E+00 1.83518859E-02 3.20701120E-05    3
-4.64664898E-08 1.70336994E-11-1.79749902E+03 1.47575576E+01                   4
[illegible]
C6H14                   C   6H  14          G   200.000  2000.000 1000.00      1
 1.65859088E+01 1.77701357E-02 1.77026482E-05-1.53531255E-08 3.20180868E-12    2
-2.73664123E+04-5.69373864E+01 5.21529155E+00 1.85496391E-02 8.35878414E-05    3
-1.03979553E-07 3.65341570E-11-2.28571406E+04 9.09501014E+00                   4
C7H16                   C   7H  16          G   200.000  2000.000 1000.00      1
 1.94402908E+01 2.01461877E-02 2.08363572E-05-1.79397710E-08 3.73807192E-12    2
-3.10416120E+04-7.00155381E+01 5.76507700E+00 2.23694911E-02 9.62177300E-05    3
-1.20671571E-07 4.25404100E-11-2.56826916E+04 9.07885165E+00                   4
C8H18                   C   8H  18          G   200.000  2000.000 1000.00      1
 2.20787675E+01 2.31535572E-02 2.32891109E-05-2.02084148E-08 4.21994846E-12    2
-3.46905487E+04-8.25366537E+01 5.90336231E+00 2.82180592E-02 1.05148036E-04    3
-1.34418151E-07 4.76816622E-11-2.84736117E+04 1.04096060E+01                   4
C10H22                  C  10H  22          G   200.000  2000.000 1000.00      1
 2.78568113E+01 2.77893918E-02 2.96539623E-05-2.54213250E-08 5.29859526E-12    2
-4.19888143E+04-1.08454827E+02 7.25836418E+00 3.48708890E-02 1.32000153E-04    3
-1.68964410E-07 6.00124394E-11-3.41035103E+04 9.74884595E+00                   4
C12H26                  C  12H  26          G   200.000  2000.000 1000.00      1
 3.33414226E+01 3.32692481E-02 3.51264323E-05-3.02217656E-08 6.30696098E-12    2
-4.94174780E+04-1.33263507E+02 8.39586803E+00 4.26752610E-02 1.56581721E-04    3
-2.01568164E-07 7.17376118E-11-3.99095568E+04 9.67842547E+00                   4
C18H38                  C  18H  38          G   200.000  2000.000 1000.00      1
 5.01453131E+01 4.86405665E-02 5.26951043E-05-4.51616383E-08 9.42477718E-12    2
-7.17116576E+04-2.09642537E+02 1.19573441E+01 6.50408289E-02 2.32622131E-04    3
-3.01464603E-07 1.07588422E-10-5.72564831E+04 8.68024248E+00                   4
END
```

Figure S8. Thermochemical data in NASA polynomial format calculated using the framework described in the main manuscript.

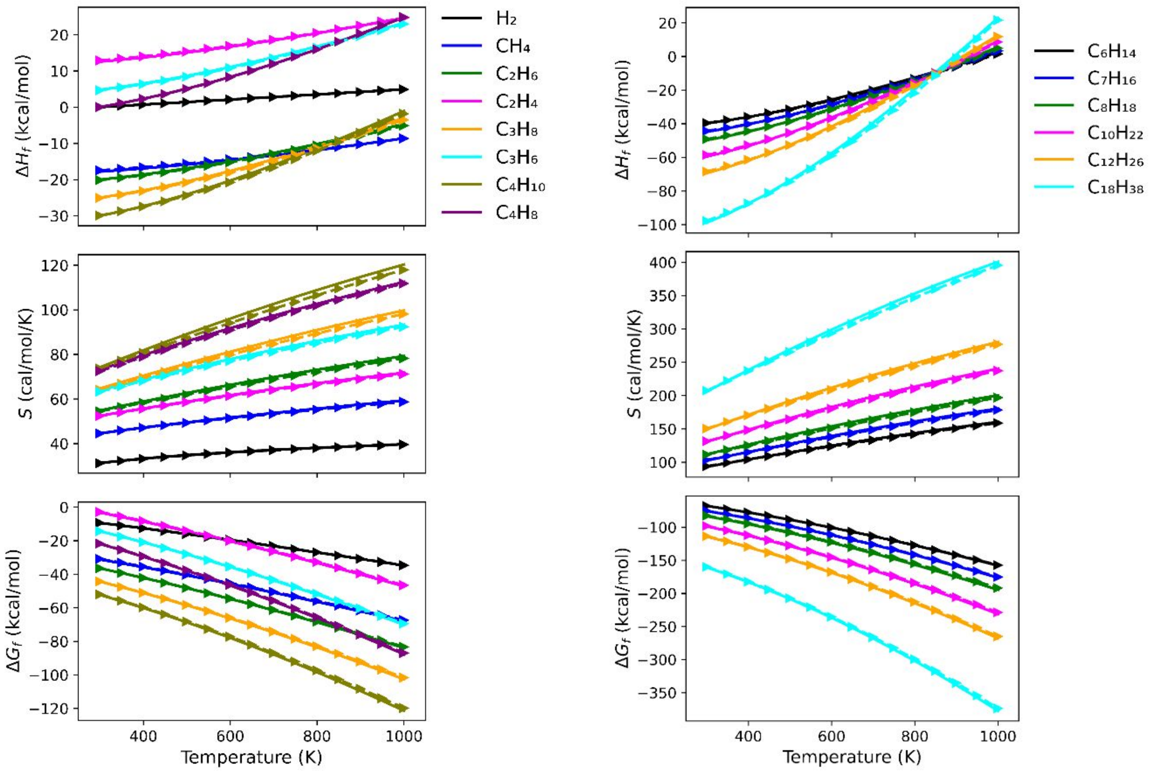

Figure S9. Enthalpy of formation (ΔH_f), entropy (S), and Gibbs free energy of formation (ΔG_f) of species considered in this work. Solid lines and dotted lines with triangle markers denote the thermochemical data from Burcat's database and from the NASA polynomials presented in Figure S8, respectively.

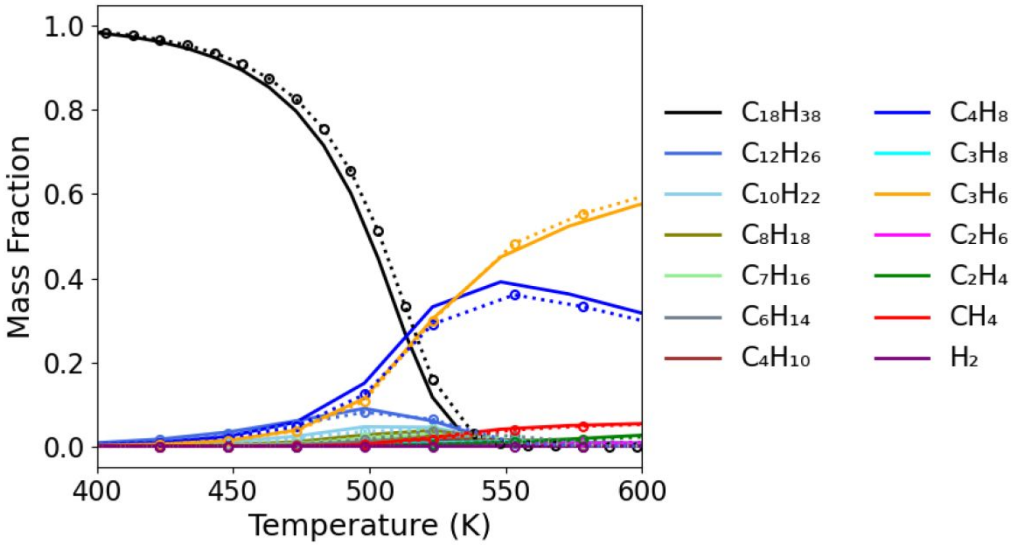

Figure S10. Equilibrium composition of octadecane decomposition in the temperature range of 400 K to 600 K using the Gibbs minimization method. Solid lines and dotted lines with circle markers denote the results using thermochemical data from the Burcat's database and DFT, respectively.

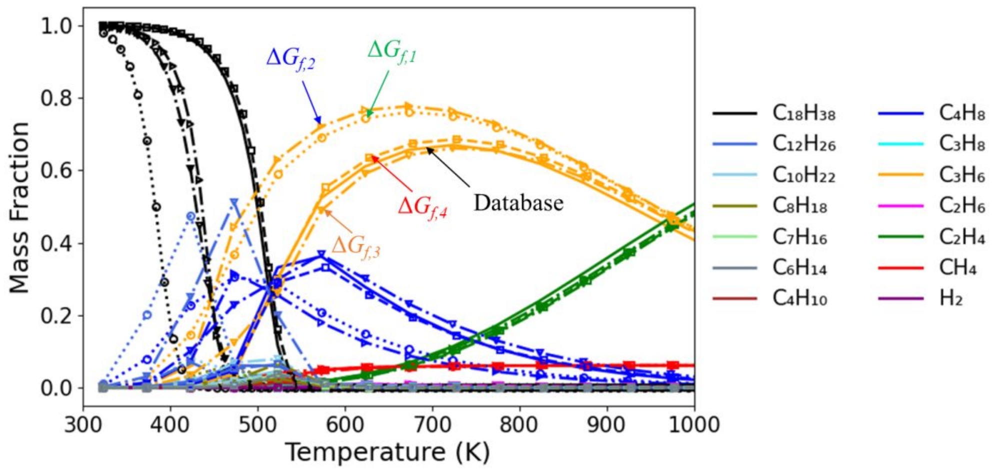

Figure S11. Equilibrium compositions of octadecane decomposition. Dotted lines with circle, triangle-right, triangle-down, and square markers indicate the simulation results using $\Delta G_{f,1}$, $\Delta G_{f,2}$, $\Delta G_{f,3}$, and $\Delta G_{f,4}$. The definition of ΔG_f can be found in Table 1 of the main manuscript. Solid lines indicate the simulation results using Burcat's database.

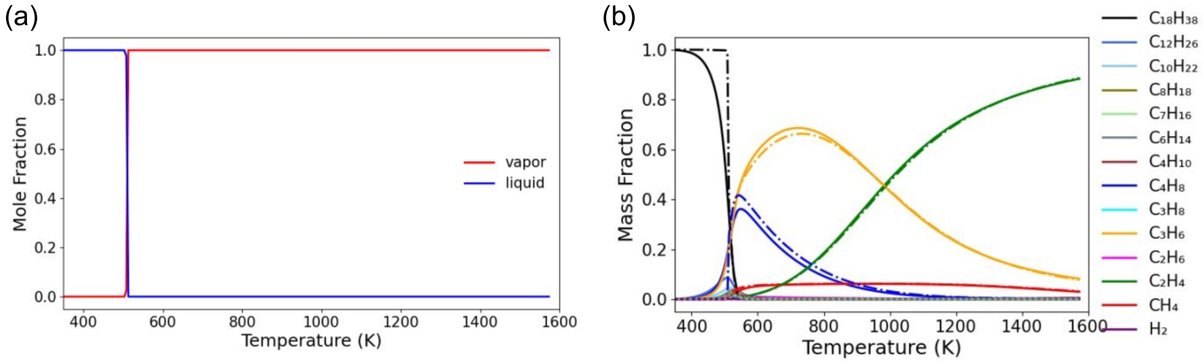

Figure S12. (a) Mole fractions of all species in vapor and liquid in chemical equilibrium simulations for a two-phase (gas-liquid) ideal mixture system using Aspen. (b) Equilibrium composition of octadecane decomposition. Solid and dotted lines represent the simulation results of a single-phase (gas) system using Chemkin and a two-phase (gas-liquid) system using Aspen, respectively. In the single-phase simulation, all species are treated as ideal gases. In the two-phase simulation, ideal models are used for both liquid and vapor phases.

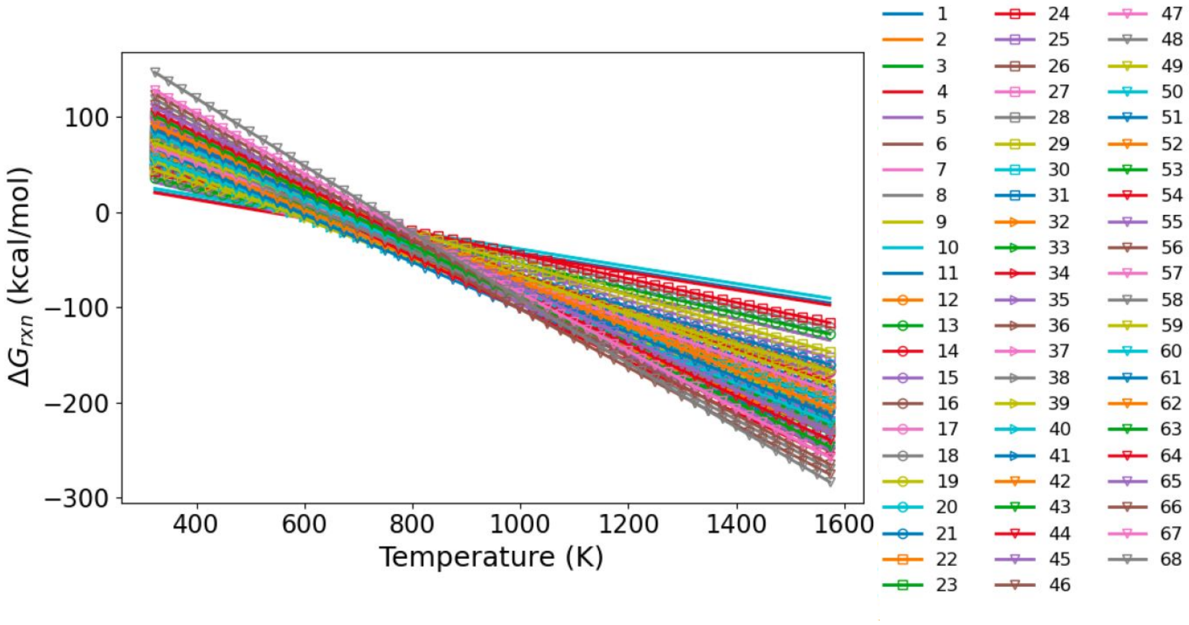

Figure S13. Gibbs free energy of reactions. The reaction indices denoted in the legend can be found in Table S1.

Table S1. Possible reactions involved in the octadecane ($C_{18}H_{38}$) decomposition. The numbers in the table indicate the number of molecules in the product side.

Index	H_2	CH_4	C_2H_6	C_2H_4	C_3H_8	C_3H_6	C_4H_{10}	C_4H_8	C_6H_{14}	C_7H_{16}	C_8H_{18}	$C_{10}H_{22}$	$C_{12}H_{26}$
1	0	0	0	0	0	0	0	2	0	0	0	1	0
2	0	0	0	0	0	0	0	3	1	0	0	0	0
3	0	0	0	0	0	1	0	2	0	1	0	0	0
4	0	0	0	0	0	2	0	0	0	0	0	0	1
5	0	0	0	0	0	2	0	1	0	0	1	0	0
6	0	0	0	0	0	2	1	2	0	0	0	0	0
7	0	0	0	0	0	4	0	0	1	0	0	0	0
8	0	0	0	0	1	1	0	3	0	0	0	0	0
9	0	0	0	0	1	5	0	0	0	0	0	0	0
10	0	0	0	1	0	0	0	1	0	0	0	0	1
11	0	0	0	1	0	0	0	2	0	0	1	0	0
12	0	0	0	1	0	0	1	3	0	0	0	0	0
13	0	0	0	1	0	2	0	0	0	0	0	1	0
14	0	0	0	1	0	2	0	1	1	0	0	0	0
15	0	0	0	1	0	3	0	0	0	1	0	0	0
16	0	0	0	1	0	4	1	0	0	0	0	0	0
17	0	0	0	1	1	3	0	1	0	0	0	0	0
18	0	0	0	2	0	0	0	1	0	0	0	1	0
19	0	0	0	2	0	0	0	2	1	0	0	0	0
20	0	0	0	2	0	1	0	1	0	1	0	0	0
21	0	0	0	2	0	2	0	0	0	0	1	0	0
22	0	0	0	2	0	2	1	1	0	0	0	0	0
23	0	0	0	2	1	1	0	2	0	0	0	0	0
24	0	0	0	3	0	0	0	0	0	0	0	0	1
25	0	0	0	3	0	0	0	1	0	0	1	0	0
26	0	0	0	3	0	0	1	2	0	0	0	0	0
27	0	0	0	3	0	2	0	0	1	0	0	0	0
28	0	0	0	3	1	3	0	0	0	0	0	0	0
29	0	0	0	4	0	0	0	0	0	0	0	1	0
30	0	0	0	4	0	0	0	1	1	0	0	0	0
31	0	0	0	4	0	1	0	0	0	1	0	0	0
32	0	0	0	4	0	2	1	0	0	0	0	0	0
33	0	0	0	4	1	1	0	1	0	0	0	0	0
34	0	0	0	5	0	0	0	0	0	0	1	0	0
35	0	0	0	5	0	0	1	1	0	0	0	0	0
36	0	0	0	6	0	0	0	0	1	0	0	0	0

37	0	0	0	6	1	1	0	0	0	0	0	0	0
38	0	0	0	7	0	0	1	0	0	0	0	0	0
39	0	0	1	0	0	0	0	4	0	0	0	0	0
40	0	0	1	0	0	4	0	1	0	0	0	0	0
41	0	0	1	1	0	2	0	2	0	0	0	0	0
42	0	0	1	2	0	0	0	3	0	0	0	0	0
43	0	0	1	2	0	4	0	0	0	0	0	0	0
44	0	0	1	3	0	2	0	1	0	0	0	0	0
45	0	0	1	4	0	0	0	2	0	0	0	0	0
46	0	0	1	5	0	2	0	0	0	0	0	0	0
47	0	0	1	6	0	0	0	1	0	0	0	0	0
48	0	0	1	8	0	0	0	0	0	0	0	0	0
49	0	1	0	0	0	3	0	2	0	0	0	0	0
50	0	1	0	1	0	1	0	3	0	0	0	0	0
51	0	1	0	1	0	5	0	0	0	0	0	0	0
52	0	1	0	2	0	3	0	1	0	0	0	0	0
53	0	1	0	3	0	1	0	2	0	0	0	0	0
54	0	1	0	4	0	3	0	0	0	0	0	0	0
55	0	1	0	5	0	1	0	1	0	0	0	0	0
56	0	1	0	7	0	1	0	0	0	0	0	0	0
57	1	0	0	0	0	2	0	3	0	0	0	0	0
58	1	0	0	0	0	6	0	0	0	0	0	0	0
59	1	0	0	1	0	0	0	4	0	0	0	0	0
60	1	0	0	1	0	4	0	1	0	0	0	0	0
61	1	0	0	2	0	2	0	2	0	0	0	0	0
62	1	0	0	3	0	0	0	3	0	0	0	0	0
63	1	0	0	3	0	4	0	0	0	0	0	0	0
64	1	0	0	4	0	2	0	1	0	0	0	0	0
65	1	0	0	5	0	0	0	2	0	0	0	0	0
66	1	0	0	6	0	2	0	0	0	0	0	0	0
67	1	0	0	7	0	0	0	1	0	0	0	0	0
68	1	0	0	9	0	0	0	0	0	0	0	0	0

References

(1) Burcat, A.; Ruscic, B. Third Millennium Ideal Gas and Condensed Phase Thermochemical Database for Combustion with Updates from Active Thermochemical Tables. ANL-05/20 and TAE 960 Technion-IIT, Aerospace Engineering, and Argonne National Laboratory, Chemistry Division **2005**.

(2) Goos, E.; Burcat, A.; Ruscic, B. Extended Third Millennium Ideal Gas Thermochemical Database with Updates from Active Thermochemical Tables. http://garfield.chem.elte.hu/Burcat/burcat.html.

(3) Petersson, G. A.; Malick, D. K.; Wilson, W. G.; Ochterski, J. W.; Montgomery, J. A.; Frisch, M. J. Calibration and Comparison of the Gaussian-2, Complete Basis Set, and Density Functional Methods for Computational Thermochemistry. *J. Chem. Phys.* **1998**, 109 (24), 10570–10579.

(4) Ghahremanpour, M. M.; Van Maaren, P. J.; Ditz, J. C.; Lindh, R.; Van Der Spoel, D. Large-Scale Calculations of Gas Phase Thermochemistry: Enthalpy of Formation, Standard Entropy, and Heat Capacity. *J. Chem. Phys.* **2016**, 145 (11).

(5) Pracht, P.; Grimme, S. Calculation of Absolute Molecular Entropies and Heat Capacities Made Simple. *Chem. Sci.* **2021**, 12 (19), 6551–6568.
